# Comprehensive comparison of enzymatic and bisulfite DNA methylation analysis in clinically relevant samples

**DOI:** 10.1186/s13148-025-01959-0

**Published:** 2025-10-03

**Authors:** Barrett Nuttall, Daniel L. Karl, Kathleen Burke, Megan Callahan, Kerrin Mendler, Pablo Cingolani, Steven Criscione, Serhiy Naumenko, Elena Bibikova, Veerendra Munugalavadla, John C. Byrd, Richard R. Furman, Jennifer R. Brown, Andrew Mortlock, Brian A. Dougherty, J. Carl Barrett, Maurizio Scaltriti, James Hadfield

**Affiliations:** 1 Early Oncology Translational Medicine, Genomics, Oncology R&D, AstraZeneca, Waltham, MA USA; 2 Early Oncology Translational Medicine, Production Informatics, Oncology R&D, AstraZeneca, Waltham, MA USA; 3 Newborn Screening Ontario, Ottawa, ON Canada; 4 Early Oncology Translational Medicine, Hematology, Oncology R&D, AstraZeneca, South San Francisco, CA USA; 5 The Ohio State University Comprehensive Cancer Center, Columbus, OH USA; 6 Division of Hematology and Oncology, Weill Cornell Medical College, New York, NY USA; 7 Department of Medical Oncology, Dana-Farber Cancer Institute, Boston, MA USA; 8 Early Oncology Translational Medicine, Oncology R&D, AstraZeneca, Waltham, MA USA; 9 Early Oncology Translational Medicine, Genomics, Oncology R&D, AstraZeneca, Cambridge, UK; 10 Precede Biosciences, Boston, MA USA

**Keywords:** Methylation, Enzymatic methylation sequencing, Bisulfite sequencing, Targeted methylation sequencing, Chronic lymphocytic leukemia

## Abstract

**Background:**

Bisulfite conversion is considered the gold standard for DNA methylation analysis, but it damages DNA and performs sub-optimally with clinical samples (e.g., formalin-fixed paraffin-embedded and circulating free plasma DNA (cfDNA)). Here we describe a comprehensive comparison of bisulfite and enzymatic methylation sequencing, using commercially available assays in clinically relevant patient samples and cell lines. We also report the first clinical enzymatic whole genome methylation sequencing (WGMS) in a cohort of patients with chronic lymphocytic leukemia (CLL). We report data from a multi-arm experiment comprising controlled reference material and clinically relevant samples to assess technical differences between enzymatic and chemical methylation conversion technologies.

**Results:**

Enzymatic methylation sequencing was highly concordant to bisulfite data but outperformed bisulfite conversion in key sequencing metrics; the enzymatic method demonstrated significantly higher estimated counts of unique reads, reduced DNA fragmentation, and higher library yields than bisulfite conversion. Enzymatic conversion produced inferior methylation array data. Although bisulfite and enzymatic methods were highly concordant, the increased quality of multiple sequencing metrics seen in the enzymatic method enabled the development of robust clinical sample pipelines including targeted sequencing in cfDNA.

**Conclusions:**

Using the enzymatic methylation sequencing methods described, we report a putative link of interleukin (IL)-15 methylation changes to acalabrutinib treatment response in a CLL clinical trial cohort (ACE-CL-001 trial, NCT02029443).

**Supplementary Information:**

The online version contains supplementary material available at 10.1186/s13148-025-01959-0.

## Introduction

Epigenetic dysregulation is a driving event in the progression of human cancers. Many epigenetic changes occur early in tumorigenesis, making epigenetic biomarkers a powerful tool in the oncologist’s armamentarium [1, 2]. Epigenetic mechanisms such as cytosine methylation, histone modifications, nucleosome and nuclear architecture, and micro-RNAs are involved in the regulation of normal cellular processes, development, and disease. Bisulfite conversion to measure DNA methylation in cancers is one of the most widely used methods for probing the epigenome, particularly at 5′-CpG-3′ sites. (The biological relevance of non-CpG methylation is less clear and is not assessed here.) However, this approach has multiple limitations, and new enzymatic methods were recently developed that may provide improved analytical methods [3–5].

Bisulfite conversion, first reported in 1992 [6], is the gold-standard DNA methylation method and has been used in multiple major epigenomics mapping efforts, including the NIH Roadmap Epigenomics Project [7], ENCODE [8], the Cancer Genome Atlas [9], and the International Human Epigenome Consortium [10]. Sodium bisulfite treatment of DNA leads to the deamination of unmethylated cytosine (C) to uracil, which is amplified in subsequent polymerase chain reaction (PCR) as thymine (T), whereas methylated cytosines (both 5-methylated cytosines [5mC] and 5-hydroxymethylated cytosines [5hmC]) are resistant to this bisulfite conversion and are amplified as usual in PCR. Bisulfite conversion leads to cytosine-to-thymine (C > T) transitions that can be detected following DNA amplification and sequencing, enabling differentiation of methylated from unmethylated cytosines at single-base resolution. Bisulfite methods cannot differentiate between 5mC and 5hmC.

Bisulfite conversion chemistry has two primary limitations: DNA damage and a reduction in base complexity. DNA damage in the bisulfite process is caused by the high temperatures and pH needed in many conversion kit methods and results in DNA degradation by depyrimidination [11], rather than long-suspected depurination [12]. Base complexity is severely reduced because bisulfite conversion affects the predominantly unmethylated cytosines in the genome; this results in sequencing libraries with a highly unbalanced nucleotide composition (the same issue affects enzymatic methods), which complicates sequencing and downstream bioinformatic analysis. Both issues are evident in the analysis of the combination of bisulfite with next-generation sequencing: whole genome bisulfite sequencing (WGBS) [13, 14]. While recent comparisons of bisulfite conversion kit methods show that not all bisulfite protocols are equally damaging, there are various sources of methodologic bias that impact the ability to detect differentially methylated regions [15–17].

Several groups have reported enzymatic methods are less damaging to DNA than chemical conversion. The APOBEC-coupled epigenetic sequencing (ACE-Seq) [3] method uses APOBEC3A enzymatic deamination of C and 5mC to uracil and thymine, respectively, with uracil’s detected as thymine’s after subsequent PCR amplification. TET-assisted pyridine borane sequencing (TAPS) [4] uses TET enzymatic oxidation of modified cytosines, both 5mC and 5hmC, followed by chemical reduction to uracil. Most recently, enzymatic methyl-seq (EM-seq) [5] uses TET2 enzymatic oxidation of modified cytosines, both 5mC and 5hmC, combined with T4-BGT enzymatic glucosylation of 5hmC, to protect against APOBEC3A deamination, which converts unmodified cytosines to uracil. In all these methods, the subsequent PCR amplification replaces uracil with thymine, leading to the same C > T transition as bisulfite conversion.

In this study, we directly compared enzymatic methylation conversion (NEBNext EM-seq; New England Biolabs, Ipswich, MA, USA [5]) with the gold-standard bisulfite conversion, using a post-bisulfite adapter tagging (PBAT) approach (EZ-96 DNA Methylation-Gold, Zymo Research, Irvine, CA, USA and Accel-NGS methyl-seq DNA library kit [hereafter referred to as BS-seq, also used generally to refer to bisulfite conversion followed by NGS, and not to be confused with the original BS-Seq method [14]], Swift Bioscience, Ann Arbor, MI, USA) for whole genome methylation sequencing (WGMS), targeted methylation panel sequencing and MethylationEPIC [18] arrays (EPIC; Illumina, San Diego, CA, USA) (Supplemental Fig. 1). These studies were completed in multiple, predominantly clinically relevant sample types: cell lines, isolated peripheral blood mononuclear cells (PBMCs), fresh frozen and formalin-fixed tissue and circulating free plasma DNA (cfDNA).

**Fig. 1:**
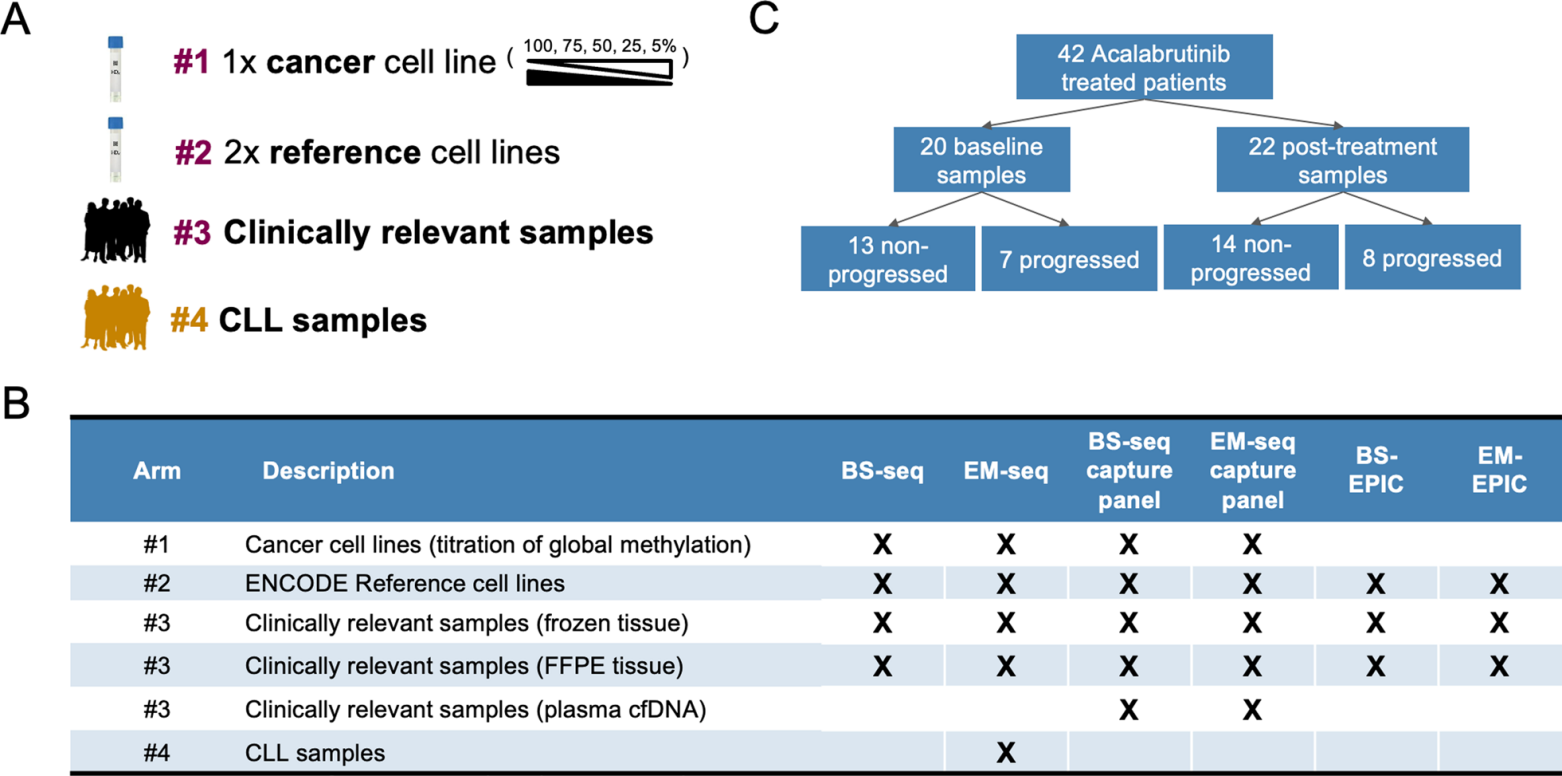
A) Samples used in study arms: Arm 1 HCT116 cell line, Arm 2 NA12878 & K562 cell lines, Arm 3 patient-derived material, and Arm 4 CLL samples. B) Data generated on each arm of the study. C) Arm 4 CLL patient clinical characteristics. CLL, chronic lymphocytic leukemia; CRC, colorectal cancer; EPIC, MethylationEPIC array; NSCLC, non-small cell lung cancer; WGMS, whole genome methylation sequencing

We also report the first clinical enzymatic WGMS in a cohort of patients with chronic lymphocytic leukemia (CLL), a malignancy for which epigenetic alterations are well characterized using bisulfite sequencing and arrays [19–23]. These bisulfite studies have shown that stages of B cell maturation [23] can be determined from methylation data and that CLL can be subtyped using these data [19]. Furthermore, bisulfite sequencing in parallel with ATAC-Seq has been used to identify chromatin accessibility and methylation-driven regulatory systems (i.e., H3K27) that may play a key role in understanding CLL development and developing treatments [24]. To explore clinical implications of this technology, bisulfite sequencing of patients with CLL treated with either chemotherapy [25] or chemoimmunotherapy [26] has also been used to show changes in methylation profiles following treatment. Recently, inhibitors of Bruton tyrosine kinase (BTK), a tyrosine kinase in the B-cell receptor pathway, have been approved for the treatment of CLL. The patients in this trial were treated with acalabrutinib, a second-generation small molecule inhibitor of BTK that binds irreversibly and prevents BTK-mediated signaling, leading to an inhibition of the growth of malignant B cells overexpressing BTK [27]. Methylation patterns have been associated with patient risk and outcome for treatment with a BTK inhibitor [22]. Chromatin immunoprecipitation sequencing (ChIP-Seq) studies investigated longitudinal changes following BTK inhibition and identified the roles of regulatory pathways in response to treatment, including treatment-induced H3K27 methylation effects on cell cycle progression [28]. Acalabrutinib was shown to have high overall response rates in CLL, but disease-free progression at 45 months of 62% of patients in the ACE-CL-001 (NCT02029443) study [29], highlighting a medical need to understand mechanisms of resistance; including those that may be driven by epigenetics.

### Results

This was a multi-arm experiment comprising reference material and clinically relevant samples to assess technical differences between enzymatic and chemical methylation conversion technologies (Fig. 1A). Arm 1 was a titration of methylation levels, which was prepared by mixing hypermethylated and hypomethylated cell line material at defined ratios to create predictable levels of genomic methylation. Arm 2 consisted of reference cell lines from the ENCODE database: NA12878 and K562 [30]. These samples are well characterized by whole genome bisulfite sequencing and represent a benchmark with which to compare our results. Arm 3 consisted of material from three patients with cancer: matched fresh frozen (FFZN) and formalin-fixed paraffin-embedded (FFPE) tumor tissue, fresh frozen normal tissue, and plasma from two disease indications—non-small cell lung cancer (NSCLC) and colorectal cancer (CRC). It is of critical importance to demonstrate that DNA methylation analysis methods are appropriate for use in FFPE tumor used for routine pathology and plasma-derived cfDNA for liquid biopsy, as these represent the most common clinical sample types.

Using the experimental samples from the multi-arm experiment described above, we compared three different high-throughput methylation assays—WGMS, targeted methylation capture, and EPIC arrays (Fig. 1B and Supplemental Table 1).

In order to examine epigenetic consequences of acalabrutinib on tumor progression in clinical samples, we performed WGMS using the best-practice conversion protocol determined from the multi-arm experiment in Arm 4 on 42 CLL samples from 22 patients (Fig. 1C) with pretreatment samples taken on cycle 1, day 1 and post-treatment samples taken at cycle 6, day 28 or at the time of relapse.

### Whole genome methylome sequencing of a methylation dilution series (Arm 1)

**Fig. 2:**
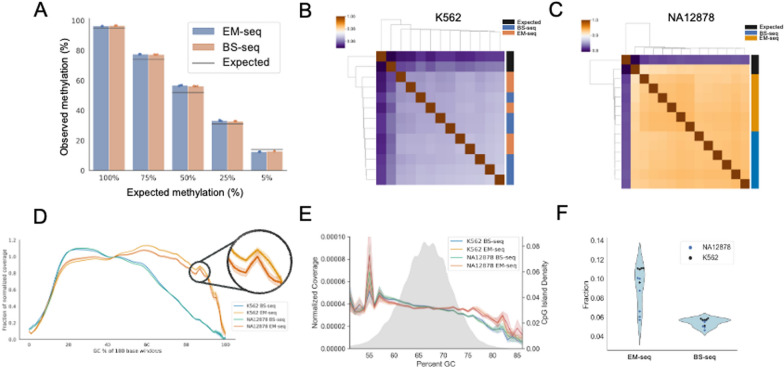
A) HCT116 DNMT1 and DNMT3b knockout cell lines diluted with in vivo methylated DNA at the indicated percentages (ns). Each dilution point includes three replicates per preparation methodology. B) K562 and C) NA12878 pairwise Pearson correlations of cytosine methylation in CpG contexts with at least 10 × coverage. D) Normalized genome coverage by %GC content in 100 bp windows*. E) Normalized coverage over CpG islands and CpG island density by GC%*. F) Fraction of Cs called in CpG islands as a fraction of total fragment pairs (p = 1.4e^−4^). *Shaded areas represent 95% CI windows; zoom shown for context. BS-seq, bisulfite cytosine conversion and next-generation sequencing; C, cytosine; EM-seq, enzymatic cytosine conversion and next-generation sequencing; GC, guanine/cytosine

Enzymatic and bisulfite treatment of DNA libraries convert unprotected cytosines to uracil through distinct reactions that may be biased depending on the methylation status of the DNA. To assess the efficiency biases of both bisulfite and enzymatic conversion, a dilution series of artificial methylation levels were created and sequenced, with three replicates per condition. Methylation titration samples were generated by blending hypermethylated and hypomethylated human control DNA to achieve specific methylation levels. Average methylation values across the genome demonstrate that both BS-seq and EM-seq methods matched closely, but not exactly, with the expected methylation values for fully methylated and unmethylated libraries (Fig. 2A; see Methods for a detailed description of the actual methylation levels expected). Lambda DNA spike-in controls indicated high conversion efficiencies using either method (Supplemental Fig. 2A). The results from the dilution series suggest that biases were not introduced during library preparation, sequencing, or computational processing.

### Whole genome methylome sequencing of cancer cell lines (Arm 2) 

To characterize global differences between bisulfite and enzymatic methods, the methylomes of two ENCODE-characterized cell lines (K562 and NA12878) were sequenced at six replicates per condition. Cytosine methylation levels in the CpG context showed strong correlation between our BS-seq and EM-seq datasets, as well as with the publicly available ENCODE bisulfite sequencing data for both K562 and NA12878 (Fig. 2B, C). Notably, the ENCODE datasets were generated using the classical pre-bisulfite adaptor tagging approach, in contrast to our internally generated data. We observed significant but modest differences between methods in conversion rates, mapping efficiency, and methylation across repeat regions (Supplemental Fig. 2B–D).

It is reported that EM-seq generates better coverage of high GC content regions than bisulfite-converted libraries [5]. While EM-seq libraries maintained higher coverage over GC-rich regions genome wide, both BS-seq and EM-seq methods displayed comparable normalized coverage over CpG islands (Fig. 2D, E). However, CpG islands were covered at substantially higher read fractions in EM-seq, with significantly higher cytosine methylation calls in CpG contexts when normalized by read count (Fig. 2F). We observed modest differences in library fragment and read lengths between the methods (Supplemental Fig. 2E, F and I). Input DNA was fragmented according to method-specific parameters (BS-seq = 350 bp, EM-seq = 250 bp), but BS-seq showed a more than twofold reduction in final library fragment size (BS-seq = 217 bp, EM-seq = 209 bp) (Supplemental Table 2). BS-seq libraries exhibited lower relative coverage over GC-rich regions due to bisulfite induced DNA fragmentation. This fragmentation leads to the depletion of unmethylated regions rich in cytosine, which results in the observed genomic bias [17]. EM-seq generated significantly higher library yields than BS-seq (489 ng vs 77 ng) using the same-input DNA amount (Supplemental Fig. 2G) and also generated significantly fewer duplicate reads (Supplemental Fig. 2H).

Together, these data demonstrate that EM-seq more efficiently captured unique methylation events in potentially biologically relevant sites such as CpG islands.

### Whole genome methylome sequencing of clinically relevant samples (Arm 3)

**Fig. 3:**
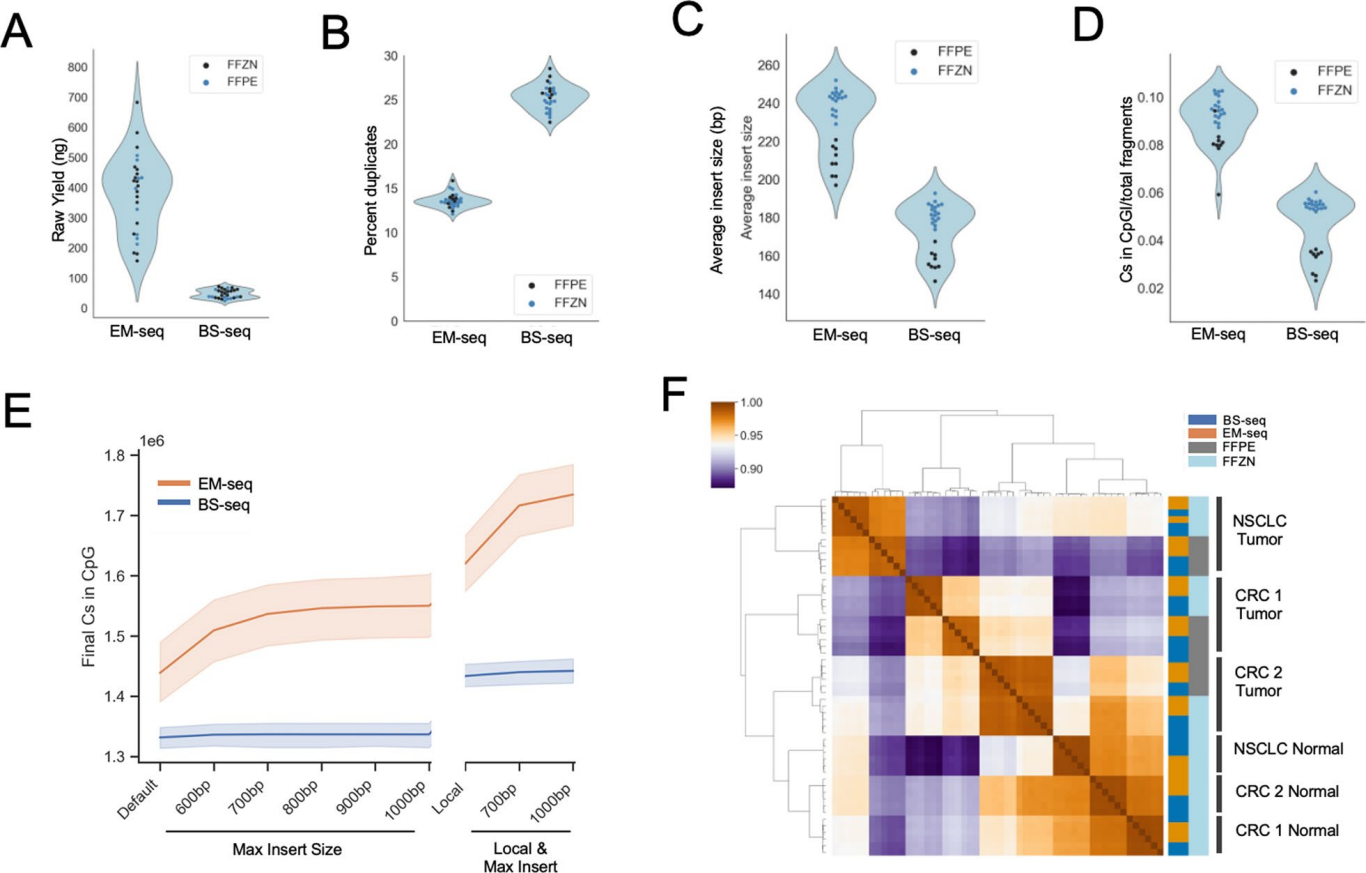
A) Library yield*. B) Percent duplicates from Bismark deduplication*. C) Average insert size using Bismark with default parameters*. D) Fraction of called cytosines in CpG Islands as a fraction of total fragments*. E) Total number of called extracted cytosines in CpG context across Bismark parameters**. F) Clustered heatmap of sample-to-sample Pearson correlations of average methylation over CpG islands. *P-value < *P* < 1e^−6^. **Shaded regions reflect 95% CI. BS-seq, bisulfite cytosine conversion and next-generation sequencing; CRC, colorectal cancer; EM-seq, enzymatic cytosine conversion and next-generation sequencing; FFPE, formalin-fixed paraffin-embedded; FFZN, fresh frozen; NSCLC, non-small cell lung cancer

Three patient tumors from two disease indications (NSCLC and CRC) were sequenced in triplicate to compare the performance of BS-seq and EM-seq conversion on patient tumor samples. In these clinically relevant samples, and similar to the cell line results reported above, EM-seq outperformed BS-seq in library yield, percentage duplicates, and library complexity (Fig. 3A and B**; Supplemental **Fig. 3A). EM-seq generated larger fragments sizes that, combined with a higher library complexity, led to a higher rate of called CpGs (Fig. 3C and D) and a larger fraction of GC-rich regions (Supplemental Fig. 3E). As seen in Arm 2, BS-seq showed a twofold reduction in final library fragment size (BS-seq = 173 bp, EM-seq = 230 bp) (Supplemental Table 2). BS-seq libraries mapped at higher rates than EM-seq libraries with default Bismark alignment parameters (82% vs 70%) despite having lower CpG coverage (Fig. 3E**; **Supplemental Fig. 3B). Increasing insert size thresholds boosted EM-seq but not BS-seq mapping (Fig. 3E) and using local alignment further increased mapping rates in both BS-seq and EM-seq libraries to comparable rates but also increased the general error rates and was less practical across many samples due to the substantial increase in computational resources required (Supplemental Fig. 3B and C). Nevertheless, default Bismark alignment parameters resulted in significantly higher coverage over CpGs in EM-seq that increased with parameter optimization (Fig. 3E).

While FFZN tissue is preferred for generating sequencing libraries, FFPE tumor biopsy or resection material remain more common sources of patient tissue. To determine whether EM-seq and/or BS-seq was robust for use with FFPE samples, patient-matched FFZN and FFPE samples were sequenced. Within each method, FFZN samples generated larger insert fragments (Fig. 3E) but otherwise appeared comparable to FFPE-derived libraries for both conversion methods. Concordance analysis of CpG island methylation averages indicated that FFZN and FFPE samples were more similar than across conversion methods (Fig. 3F). Differentially methylated region analysis performed using three different methods identified no significantly differentially methylated CpG islands between FFZN and FFPE samples in either EM-seq or BS-seq (**Supplemental **Fig. 3D). These data indicate that both EM-seq and BS-seq methods are robust for monitoring changes in methylation on FFPE samples, even though enzymatic methods captured a slightly larger fraction of GC-rich regions (Supplemental Fig. 3E).

### Targeted methylome sequencing

WGMS is often cost prohibitive to reach substantial coverage across the genome for large numbers of patients. Most of the captured cytosine methylation events are filtered before downstream analyses based on genomic location or distance from a gene, transcript, or CpG island. As such, methylation capture panels provide a cost-effective method for high-coverage interrogation of targeted genomic regions of interest.

**Fig. 4:**
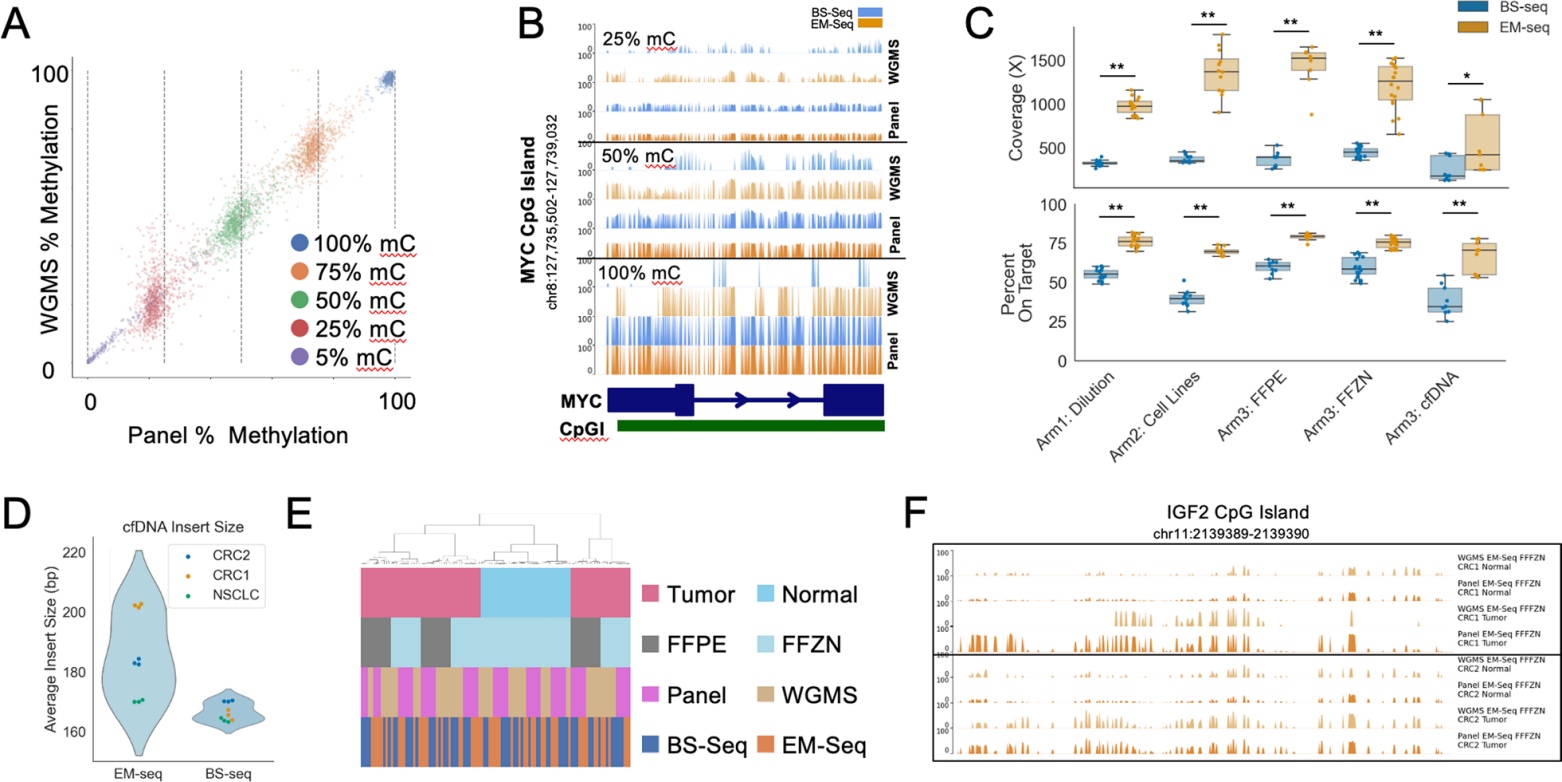
A) Average percent methylation in panel regions in WGMS vs Capture sequencing for Arm1 EM-seq dilution samples. (p < 1e-4,*R* = 0.99, Pearson Correlation). B) Percent methylation for Arm1 samples at indicated methylation dilutions across CpGs with >  = 10 × CpG coverage at a CpG Island within MYC. C) Coverage and On Target Percentage for panel capture sequencing. (*p = 0.03, **p < 1e-4) for all EM v BS except cfDNA. D) Mean insert size for panel capture cfDNA. (p = 3.9e-3, Wilcoxon signed-rank). E) Clustering samples by Pearson correlation across panel region average methylation. F). Percent methylation by CpG across the IGF2 CpG Island for CpGs with 10X coverage or greater

To validate the methylation capture approach, we sequenced Arms 1, 2 & 3 using a custom-designed Twist Bioscience methylation hybridization capture panel (see **Targeted methylation panel design** in Methods). We first benchmarked expected results from our Arm 1 titration samples and determined that sequenced libraries were accurately capturing methylation fraction across multiple hyper- and hypomethylated substrates (Fig. 4A and B). Despite dropout at a small number of targets (within manufacturers guidelines), we observed high coverage across all samples compared (Fig. 4C). Mapping rates were similar across sample types but generally lower for BS-seq (Supplemental Fig. 4A). Additionally, without unique molecular identifiers (which were unavailable at the time), libraries were deduplicated according to start and end position of the read pairs, which likely leads to loss of useful biological duplicates over high-coverage panel regions. EM-seq generated significantly higher coverage and on-target rates over panel regions compared with BS-seq (Fig. 4C). Input DNA in the Arm 3 cfDNA was not fragmented for either library-prep method, but BS-seq libraries were consistently smaller than EM-seq libraries (Fig. 4D; Supplemental Table 2). Additionally, since cfDNA conversion occurs without a library-shearing step, we determined that EM-seq preserves larger fragments (and by extension more cytosines) for sequencing than BS-seq. The larger average insert size of cfDNA in the EM-seq libraries was due to the conservation of dinucleosome and trinucleosome fragments (Supplemental Fig. 5).

**Fig. 5:**
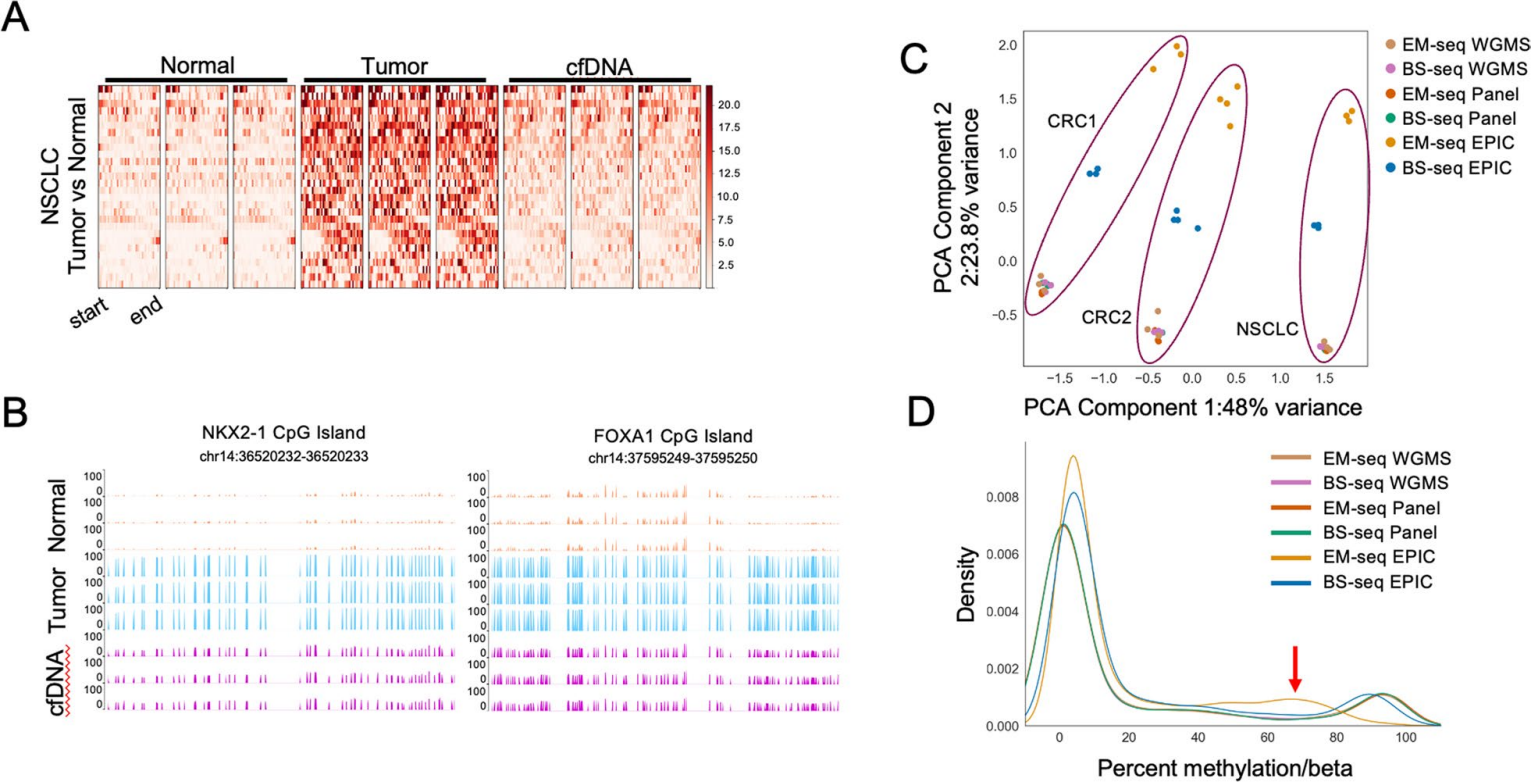
A) Heatmap of differentially methylated regions hypermethylated in tumor vs normal samples. B) Average methylation across two CpG islands in panel capture-sequencing from cfDNA of a NSCLC patient. C) PCA plot of Arm 3 samples from EPIC, panel, and WGMS. D) Density plot of panel regions by average percent methylation (NGS) or normalized beta values (EPIC). BS-seq, bisulfite cytosine conversion and next-generation sequencing; cfDNA, circulating cell-free plasma DNA; CRC, colorectal cancer; EM-seq, enzymatic cytosine conversion and next-generation sequencing; EPIC, MethylationEPIC array; NGS, next-generation sequencing; NSCLC, non-small cell lung cancer; WGMS, whole genome methylation sequencing

To determine whether targeted methylation capture generates comparable methylation data to WGMS, methylation at common genomic regions were aggregated and correlated by sample. Average methylation across panel regions was highly concordant and clustered by sample across sequencing methods (Fig. 4E). These patterns were observed for both EM-seq and BS-seq libraries generated from either FFPE or FFZN samples (**Supplemental **Fig. 4B–E). Additionally, we observed increased dropout of sequenced regions in WGMS compared to panel sequencing, driven by lower coverage across the targeted regions in the former (Fig. 5).

### Tissue:Plasma DMR Results

The custom Twist Bioscience methylation panel targeted cancer-relevant CpG islands that may act as biomarkers for tracking disease progression or for identifying putative cancer-driving events in patients. For three patients over two disease indications, EM-seq capture panel libraries were used to compare tissue from tumors and matched normal tissue. While the majority of panel regions were unchanged in tumor and normal samples, differentially methylated panel regions were readily identified between matched tumor and adjacent normal technical replicates in all three patients (Fig. 5A**; Supplemental **Fig. 6A and 6B), including identification of known hypermethylated regions in tumor samples; additionally, we saw low variation in panel coverage across the replicate samples (Fig. 5B).

**Fig. 6:**
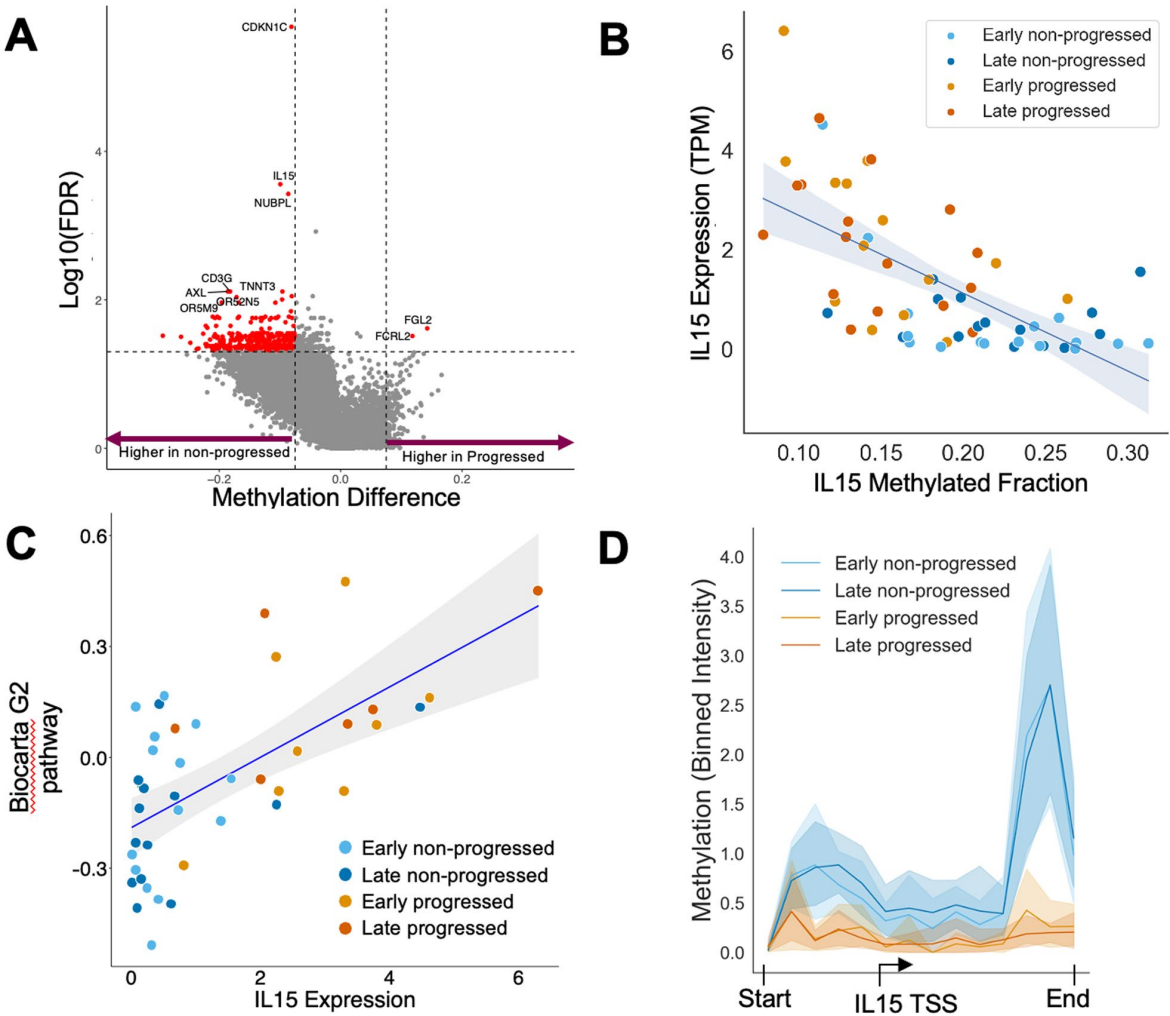
A) Volcano plot highlighting significant methylation differences in TSS regions of progressed vs non-progressed patients following treatment. B) Correlation of *IL15* methylation with expression from bulk RNA-Seq data from corresponding samples (*R* = -0.73, p < 1e-6). C) *IL15* expression correlates with the Biocarta G2 pathway, showing a significant relationship between *IL15* expression and cell cycle signaling (*R* = 0.63, p = 1e-5). D) Methylation signal profile across a CpG island at the *IL15* TSS. Line represents the average methylation of each group with shaded regions reflecting 95% CI. TSS, transcriptional start site

Over cancer-relevant panel regions, most patient-matched cell-free circulating DNA collected from peripheral blood was more similar to normal samples than tumor samples. However, there were several regions that were differentially hypermethylated in tumor samples compared with normal samples that were also significantly hypermethylated in cfDNA compared with normal samples. Intriguingly, in one patient, two cancer-related CpG islands known to be silenced in advanced NSCLC (NKX2.1 and FOXA1) were methylated in tumor and cfDNA but not in normal samples or in cfDNA of CRC patients (Fig. 5B). These genes are reported to be activated in early disease and silenced as tumors transition to advanced disease [31, 32]. These results suggest that targeted capture methylation sequencing has the potential to identify and monitor solid tumor progression from circulating cell-free DNA.

### EPIC Array Results

Microarray analysis of DNA methylation provides another well-used and cost-effective method for high-coverage interrogation of prespecified CpGs of interest. The EPIC array quantitatively interrogates > 850,000 methylation sites and has been widely used in various samples [18].

We compared the performance of enzymatic versus bisulfite cytosine conversion, using the same methods used in EM-seq and BS-seq, on the widely used EPIC arrays. FFPE samples showed a poorer conversion than FFZN for both enzymatic and bisulfite methods and were excluded from downstream analysis. In both unbiased principal component analysis and pairwise Pearson correlations, bisulfite EPIC array data clustered more closely with matched WGMS, and panel capture results than enzymatic EPIC data across CpGs covered in the panel regions (Fig. 5C). Bisulfite and enzymatic EPIC data clustered by sample rather than by conversion method or assay type. We also observed that enzymatically converted samples showed incomplete conversion by EPIC QC (**Supplemental **Fig. 6C) and right-shifted beta signal from methylated probes than bisulfite-converted samples, which more closely matched methylation results from sequenced samples (Fig. 5D**, arrow indicates EM-seq EPIC**). Together, our data suggest that enzymatic conversion may be less conducive to EPIC array-based methylation technologies, but that this underperformance of enzymatic conversion on EPIC arrays requires a more detailed investigation by array users.

### Whole Genome Methylome Sequencing of CLL

To demonstrate the clinical utility of EM-seq, we profiled a cohort of patients with CLL from the ACE-CL-001 trial treated with acalabrutinib monotherapy (NCT02029443). Patient samples were collected at baseline and following acalabrutinib monotherapy, either at 6 months after the start of treatment (cycle 6, day 28) or at the time of progression (Supplemental Table 3). All comparisons in this study are made between progressed and non-progressed patients either at baseline or following treatment to ensure that the percentage of B cells within the samples did not affect detected methylation differences. B cell percentages between progressed and non-progressed patients were not significantly different at either time point (data not shown). Methods were first validated in a set of 6 CLL samples with one sample sequenced in triplicate with varying PCR cycles (6 cycles, 5 cycles, and 4 cycles). The samples were of high quality and showed > 99% conversion efficiency and separation based on promoter methylation.

Analysis of the post-treatment samples revealed that the non-progressed patients had higher methylation of genes including *IL15*, *CDKN1C,* and *AXL* compared with progressed patients (Fig. 6A). These genes were previously shown to be related to cell cycle, proliferation, survival, or response to BTK inhibition in CLL [33–37]. All three genes highlighted above were also significantly different between the progressed and non-progressed patients at baseline. Using matched RNA-Seq, we showed that *IL15* methylation was significantly negatively correlated with *IL15* expression, as expected with higher DNA methylation and decreased expression (Fig. 6B). IL15 is a proinflammatory cytokine that has downstream signaling targets in the nuclear factor kappa B pathway, similar to BTK, indicating potential alternative methods of signaling that would be less affected by BTK inhibition. Biocarta pathway analysis identified three primary correlations, the G2 pathway (R^2^ = 0.63; Fig. 6C), the cell cycle pathway (R^2^ = 0.59), and the G1 pathway (R^2^ = 0.58), indicating that cell cycle signaling is correlated with *IL15* expression. While *CDKN1C* and *AXL* are also regulators of the cell cycle, and both showed significantly more methylation in non-progressed patients, these increases in methylation did not correlate with RNA-Seq-based expression (Supplemental Fig. 7C,D). One hypothesis regarding these data is that *IL15* methylation may be transcriptionally regulating the pathway, while *CDKN1C* and *AXL* may have alternative effects, which also result in increased cell cycle signaling. A close examination of the *IL15* methylation signal at single-base resolution identified a small region within the Il15 transcriptional start site (TSS) at a CpG island with significantly higher methylation in non-progressed patients (Fig. 6D).

## Discussion

In this study, we aimed to determine the impact of the new EM-seq (enzymatic cytosine conversion) method on the quality of DNA methylation analysis in clinically relevant samples. Our primary goal, to determine the “best” assay for clinical samples by comparing library preparation methods using BS-seq and EM-seq and identifying important differences, was met.

Together, results from the multi-arm WGMS experiment indicate that both chemical (BS-seq) and enzymatic (EM-seq) methods perform well on cell lines and in clinically relevant samples. Both methods produce highly concordant data, and we observed less dramatic differences than previous reports [5]. Nevertheless, Em-seq captured a larger fraction of GC-rich regions (Supplemental Fig. 3E), including CpG islands in all experimental arms. It also generated more complex libraries per ng of input DNA, resulting in fewer duplicate reads. For these reasons, we determined that enzymatic conversion is preferable in a clinical setting with potentially limited sample availability.

Our results confirm the challenges of bisulfite conversion as demonstrated by reduced yield of sequencing libraries and shorter average insert sizes, presumably because of the DNA damage caused during conversion. EM-seq generated significantly more library than BS-seq (Fig. 3A), had significantly reduced read duplication (Fig. 3B), and significantly higher estimated counts of unique library fragments (Supplemental Fig. 3A). This conservation of library complexity is at least partially due to a conversion process that induces less DNA fragmentation. This was evidenced by a much smaller reduction in observed versus expected final library sizes in all experimental arms (Supplemental Table 2). Additionally, the use of cfDNA in Arm 3 enables a true “apples-to-apples” comparison, as no additional shearing was performed during library preparation. Here we saw preservation of the di- and trinucleosomal peaks (often seen in cfDNA samples) in EM-seq enzymatically converted libraries that were almost absent in the bisulfite-converted BS-seq libraries (Fig. 4D and Supplemental Fig. 5), presumably because of additional DNA fragmentation of longer molecules. Additionally, the quantitative difference we saw in final library yields between BS-seq and EM-seq suggests that enzymatic methods may be a better fit for clinical workflows. This is primarily driven by the need for high-depth sequencing of cfDNA, which requires consistently high library yields from plasma samples if these methods are to be used routinely in the clinic. Importantly, none of the results presented here were affected by DNA input, as we used the same 50 ng and same 6 cycles of PCR across both EM-seq and BS-seq samples. We noted that EM-seq had a more laborious protocol workflow than BS-seq, which was also reported by Morrison et al. [38]. The additional clean-up steps and incubations required make this method less amenable to single-day automation of library preparation.

We did not see as significant a difference in overall performance of EM-seq versus BS-seq in sequencing results, as was reported by Vaisvila et al. [5], which may be due to the different bisulfite protocols chosen for comparison. Vaisvila et al. performed bisulfite conversion before adapter ligation to parallel their enzymatic conversion protocol. However, bisulfite treatment of adapter-ligated sequencing libraries are suboptimal, as any DNA damage leads to unamplifiable molecules and a lower yield, and due to the resulting need for increased PCR cycles, which lowers library quality [39]. This reduced library quality leads to an increase in overall bias, particularly a reduction in unmethylated cytosine-rich fragments, which are disproportionately lost in the final library amplification [17]. Our results, which compare the “best” bisulfite method, still confirm the improvements reported by Vaisvila et al., although our bisulfite data still demonstrate that this method, used by many researchers, can produce high-quality data. For some applications (eg, ctDNA or single cell), the reduction in DNA fragmentation from enzymatic conversion and the increase in library yields are likely to render this the current method of choice.

We saw that the computed values of percentage methylation in the Arm 1 dilution series matched closely, but not exactly, with expected values. The controls available are described as methylated and non-methylated (see Methods) but are derived from a double knockout, DNMT1 (-/-) and DNMT3b (-/-), HCT116 cell line that has low-level rather than zero DNA methylation [40]. This meant that our expected 100%, 75%, 50%, 25%, and 0% mixes were observed at 95%, 74%, 52%, 34%, and 14% methylation (Fig. 2A). Additionally, the values observed varied when analyzing targeted sequencing methylation data, where we saw 98%, 74%, 48%, 22%, and 3% methylation (Fig. 4A). This is presumably because we targeted regions that are critically methylated in cancer. However, in both cases, EM-seq and BS-seq were highly concordant in the estimation of percent methylation.

While our study identified several advantages of using enzymatic methods for clinically relevant samples for sequencing experiments, our results may suggest caution in the use of enzymatic preparation for array-based experiments (that were optimized for bisulfite-converted DNA). Our data indicated that enzymatic and bisulfite fragments appear to bind unmethylated probes efficiently, but in some clinical samples enzymatic-converted libraries showed depressed signal from methylated fragments. We speculate that one potential explanation may be that the bulk adduct that protects methylated cytosines from deamination (5 gm C) also hinders the isothermal amplification step and biases the resulting libraries by under-representing the methylated fraction. There may be other explanations that are similarly beyond the scope of this study to address, and we would suggest caution in switching from recommended EZ DNA Methylation Kit bisulfite method.

Other groups have compared DNA methylation sequencing methods. Notably, and as previously mentioned, the 2018 evaluation of bisulfite sequencing library preparation methods by Olova et al. highlighted the bisulfite conversion itself as the largest source of biases [17]. Two groups have recently compared enzymatic to bisulfite sequencing. A large multiplatform, multisite evaluation and benchmarking of cytosine modification analysis technologies by the FDA’s Epigenomics Quality Control group [41] reported high overall concordance across five WGMS methods including EM-seq and the same BS-seq as reported here. However, all data were generated from a high-quality Genome in a Bottle reference cell line DNA [42], meaning that some of their conclusions may not be relevant for the suboptimal clinical samples focused on here. Morrison et al. [38] compared four methods including EM-seq and BS-seq and reported similar findings to the results presented here; higher-quality libraries with larger insert sizes and higher library complexity. Morrison et al. used FFZN human fallopian tube samples, which, although more likely to reflect DNA used in research studies, still do not allow performance assessment in clinically relevant tissues.

Our study has several limitations. Firstly, we did not complete an apples-to-apples comparison as did Vaisvila et al. by substituting chemical for enzymatic conversion while using the same parameters for every other step. Rather, we chose a post-bisulfite adapter–tagging (PBAT) [39] protocol as the “best” bisulfite method we could identify and benchmarked this against the optimized protocol for EM-seq. PBAT methods reduce the impact of DNA damage caused by bisulfite chemistry through adapter ligation after conversion (**Supplemental **Fig. 1). It remains to be seen whether enzymatic conversion benefits from being performed after adapter ligation in a PEmAT (post-enzymatic conversion adapter tagging) approach. However, the quality of the EM-seq data reported here would suggest otherwise, and protocol or automation considerations may direct the further development of EM-seq by users. While we did not exhaustively compare computational methods, as done by Foox et al., who also characterized methylation in CHG and CHH contexts, [41], we did optimize the performance of our bioinformatics pipeline. For these analyses, we chose a conservative read-mapping approach using a standard Bismark pipeline that has been well validated on clinical samples. Other computational methods proved to be more efficient but required substantial post-alignment filter optimization that was too significant to consider. Lastly, we did not consider comparison to long-read technologies that can profile DNA methylation “natively” without chemical modification. Whist the proof of concept was published almost a decade ago [43, 44], comparisons of the two dominant technologies have been published [45], and the most recent data demonstrate the opportunities that native methylation analysis, in a long-read or short-read cfDNA context, may bring [46, 47]; these methods are still not widely used in a clinical setting and were not included here.

Despite these limitations, our results demonstrate the superior performance of enzymatic cytosine conversion over bisulfite. While EM-seq and BS-seq both produce high-quality WGMS data, the increased quality of multiple sequencing metrics and final sequencing library yield of EM-seq offer easier routes to targeted methylation approaches. The data generated from the custom Twist Bioscience panel designed for this study showed how deep sequencing of highly complex libraries can enable analysis of FFPE and cfDNA samples. Future studies of deep methylation sequencing in tissue and cfDNA are likely to identify novel mechanisms of therapeutic resistance and tumor heterogeneity. Localized changes in DNA methylation were identified in many different cancer types, raising the possibility that tracking methylation in patient samples may serve as a biomarker for early detection [48], treatment response, and/or tumor progression [49]. We describe a novel epigenetic signature in CLL that correlates with progression on acalabrutinib monotherapy. These results, from the first reported enzymatic WGMS in a clinical cohort, and which need validation in larger datasets, suggest a potential novel mechanism of resistance to BTK inhibition through decreased methylation and increased expression of *IL15*. Our pathway analysis identified a similar connection of response to BTK inhibition with cell cycle activity in CLL as seen in previous literature, where inhibition of the cell cycle was shown to be a primary effect of ibrutinib [50], and mechanisms of resistance to BTK inhibition can be tied to exit from the cell cycle [28] or insufficient inhibition of the cell cycle [51]. Our data suggest that *IL15* methylation may be transcriptionally regulating the pathway while *CDKN1C* and *AXL* may have alternative effects which also result in increased cell cycle signaling. We speculate that this could reflect a novel *IL15* regulatory region controlled in part by DNA methylation and would be of interest to explore in future studies. Although IL15 has been used in studies to understand the impact of BTK inhibition in CLL, this is the first time that DNA methylation of *IL15* has been shown to be a potential marker of progression/resistance to acalabrutinib treatment. The identification of cell cycle markers that are being affected by methylation may indicate potential opportunities for future combination treatments, including PI3K inhibitors [35] to disrupt downstream IL15 signaling. Verification of these findings could result in future combination therapies to perturb the epigenetics of acalabrutinib resistance, such as acalabrutinib with an IL15 inhibitor [52] or IL15 pathway inhibitors, such as PI3K and STAT5 inhibitors [35]. The identification of a potential methylation-driven mechanism of resistance to acalabrutinib indicates that epigenetic differences should be considered when investigating clinical biomarkers of response to BTK inhibition. Future studies will need access to the right patient samples pre- and post-therapy samples to understand the impact of therapy on the methylome more broadly. Our data and results will enable researchers interested in using these technologies to make informed choices for their DNA methylation studies.

## Conclusion

Our comprehensive comparison of enzymatic cytosine-conversion methods demonstrates that EM-seq generates higher-quality and higher-yielding libraries than bisulfite-converted libraries, with subtle, but important, effects on differential methylation analysis. Using the enzymatic methods described, we link interleukin (IL)-15 methylation changes to acalabrutinib treatment response in a CLL clinical trial cohort (ACE-CL-001 trial, NCT02029443) and highlight a possibly new and novel epigenetic mechanism of resistance, which, if validated in external cohorts, opens up new therapeutic combination opportunities.

## Methods and materials

### Wetlab methods

#### Sample acquisition

Materials from three individuals with matched FFZN tumor tissue, FFZN normal tissue, FFPE tumor tissue, and plasma material were fully consented by Indivumed Gmbh and commercially acquired for this research study. Human biologic samples purchased for this study were fully consented for all analyses. High-quality cell line DNA from K562 (DD2011, Promega Corporation, Fitchburg, WI, USA) and NA12878 (NA12878, Coriell Institute for Medical Research, Camden, NJ, USA), as well as human methylated DNA and human non-methylated DNA derived from HCT116 (D5014, Zymo Research, Irvine, CA, USA) were purchased as control material. PBMCs from the ACE-CL-001 trial were isolated from patient blood by a central laboratory and preserved as frozen cell pellet containing between 1 and 5 million cells. Twenty-two patients had post-treatment samples, and a subset of 20 also had paired baseline samples. The collection of CLL samples used in this research was approved by an institutional review board at each clinical site and conducted in accordance with the principles expressed in the Declaration of Helsinki. Written informed consent for participation in the study, including the collection, use, and sharing of data, was obtained from all patients under the NCT02029443 IRB approved protocol. All patients in the ACE-CL-001 trial were fully consented for exploratory analyses.

#### DNA extraction

DNA from FFZN tissue and PBMCs was extracted with the Mag-Bind Blood and Tissue DNA HDQ 96 Kit (M6399, Omega Bio-tek, Norcross, GA, USA). DNA from FFPE tissue was isolated with the Mag-Bind FFPE DNA Kit (M6958, Omega Bio-tek). CfDNA from plasma was isolated with the Mag-Bind cfDNA Kit (M3298, Omega Bio-tek). All isolations were performed on a Kingfisher Flex instrument (5,400,630, ThermoFisher Scientific, Spotswood, NJ, USA), utilizing scripts provided by Omega Bio-tek Inc. All extracted and procured DNA was quantified using the KAPA Human Genomic DNA Quantification Kit qPCR assay (07960603001, Roche Molecular Systems, Pleasanton, CA, USA) on a QuantStudio 7 Real-Time PCR System (4,485,701, ThermoFisher Scientific).

#### RNA extraction

RNA from PBMCs was extracted with the RNeasy Mini kit (74,106, QIAGEN, Germantown, MD, USA) with on column DNase1 digestion (79,254, QIAGEN, Germantown, MD, USA). All extracted RNA was quantified with the TapeStation RNA assay (5067–5576, Agilent Technologies, Santa Clara, CA, USA) on a 4200 TapeStation System (G2991BA, Agilent Technologies, Santa Clara, CA, USA).

#### Methylation dilution series production

Methylation titration samples were created by blending the Human HCT116 DKO Methylated DNA (D5014-1, Zymo Research Corp., Irvine, CA, USA) and Human HCT116 DKO Non-Methylated DNA (D5014-2, Zymo Research Corp., Irvine, CA, USA) material together. These Methylated and Non-Methylated controls were quantitated by the Roche KAPA Human Genomic DNA Quantification Kit qPCR assay (07960603001, Roche Molecular Systems, Pleasanton, CA, USA) on a QuantStudio 7 Real-Time PCR System (4,485,701, ThermoFisher Scientific).

The concentration values obtained from the Human Genomic DNA Quantification Kit were used to calculate the required mass of each control needed to achieve the desired methylation levels. The controls were then blended together by mass to create specific methylation levels of 100%, 75%, 50%, 25%, and 5% methylation.

These blends were created with the assumption that the Human HCT116 DKO Non-Methylated cell line, which contains knockouts for DNA methyltransferases DNMT1 and DNMT3b, would have < 5% methylation, as noted in the publicly available documentation for these products.

After obtaining the initial WGMS results, it was discovered that the Non-Methylated control reported a higher methylation value than anticipated. Upon further investigation, Zymo Research Corp confirmed that the Human HCT116 DKO Non-Methylated cell line, from which the DNA is derived, has 14% global methylation and the Human HCT116 DKO Methylated DNA has 95% global methylation. The methylation levels of the titration samples were then recalculated based on these corrected values and were found to be consistent with the values obtained from the initial WGMS analysis. The corrected methylation values for the titration samples were determined to be 95%, 74%, 52%, 31%, and 14% methylation, reflecting the revised understanding of the controls’ methylation status.

#### Whole genome bisulfite sequencing library preparation

Whole genome bisulfite sequencing libraries were prepared using the EZ-96 DNA Methylation-Gold MagPrep (D5042, Zymo Research) in conjunction with the Accel-NGS Methyl-Seq DNA Library kit (30,096, Swift Bioscience, Ann Arbor, MI, USA), following the manufacturer’s protocols. Libraries were prepared from 50 ng of sample DNA and an additional 2 ng of unmethylated lambda DNA. All samples were sheared to 350 bp using a Covaris M220 ultrasonicator (500,295, Covaris, Woburn, MA, USA). Post-conversion desulfonation washes were performed on a Kingfisher Flex instrument (ThermoFisher Scientific) utilizing a custom-developed script. All libraries proceeded through six cycles of amplification and were quantified with the TapeStation D1000 assay (5067–5582, Agilent Technologies, Santa Clara, CA, USA) on a 4200 TapeStation System (G2991BA, Agilent Technologies). Samples in Arm 1 were prepared with 3 replicates, Arm 2 were prepared with 6 replicates and Arm 3 were prepared with 3 replicates.

#### Whole genome enzymatic methylation sequencing library preparation

Whole genome enzymatic methylation sequencing libraries were prepared using the NEBNext Enzymatic Methyl-Seq Kit (E7120L, New England Biolabs, Ipswich, MA, USA), following the manufacturer's protocol. Libraries were prepared from 50 ng of sample DNA and an additional 2 ng of unmethylated lambda DNA. DNA from FFZN tissue, FFPE tissue, and cell line material were sheared to 250 bp. DNA from PBMCs was sheared to 300 bp. All shearing was performed with a Covaris M220 ultrasonicator. All libraries proceeded through six cycles of amplification and were quantified with the TapeStation D1000 assay on a 4200 TapeStation System (Agilent Technologies). Samples in Arm 1 were prepared with 3 replicates, Arm 2 were prepared with 6 replicates, and Arm 3 were prepared with 3 replicates.

#### Targeted methylation library preparation

Prior to targeted methylation capture, libraries prepared with the Accel-NGS Methyl-Seq DNA library were processed through four or five additional cycles of amplification using the KAPA HiFi HotStart ReadyMix system (7,958,935,001, Roche Molecular Systems). Libraries prepared with the NEBNext Enzymatic Methyl-Seq Kit were of high enough concentration that no additional amplification was required before capture. Samples were multiplexed together in pools consisting of 9 to 15 libraries, with each capture reaction containing a total of 1200 to 1500 ng of library. Hybridization capture was performed with a custom Twist Bioscience targeted methylation panel (see below for ***Targeted Methylation Panel Design*** parameters) and the Fast Hybridization and Wash Kit (101,174, Twist Bioscience, South San Francisco, CA, USA). The manufacturer’s protocol was followed with an adjustment of the wash buffer by 1 degree to 63°C. Enriched libraries proceeded through 10 or 11 cycles of post-capture amplification using the KAPA HiFi HotStart ReadyMix system (Roche Molecular Systems) and were quantified with the TapeStation D1000 assay on a 4200 TapeStation System (Agilent Technologies). Samples in Arm 1 were prepared with 3 replicates, Arm 2 were prepared with 6 replicates, and Arm 3 were prepared with 3 replicates.

#### RNA sequencing library preparation

RNA library sequencing preparations were performed using two separate methodologies. Preparations were performed with either the KAPA RNA HyperPrep Kit with RiboErase (KK8561, Roche Diagnostics Corporation, Indianapolis, IN, USA) or the KAPA Stranded mRNA-Seq Kit (KK8421, Roche Diagnostics Corporation, Indianapolis, IN, USA), following the manufacturer's protocols. Libraries were prepared from up to 150 ng of sample RNA and ERCC Spike-In controls (4,456,740, Thermo Fisher Scientific, Waltham, MA, USA) were added to samples prepared with the KAPA RNA HyperPrep Kit with RiboErase. All libraries were quantified with the TapeStation D1000 assay (5067–5582, Agilent Technologies, Santa Clara, CA, USA) on a 4200 TapeStation System (G2991BA, Agilent Technologies).

#### Sequencing

Sequencing of whole genome bisulfite and enzymatic methylation libraries was performed on a NovaSeq 6000 instrument (20,012,850, Illumina, San Diego, CA, USA) utilizing an S4 flowcell and 150-base, paired-end reads (20,028,312, Illumina) with 10% PhiX control library (FC-110–3001, Illumina). Sequencing of the targeted methylation libraries was performed on a NovaSeq 6000 instrument utilizing an S1 flowcell and 150 paired-end reads (20,028,312, Illumina) with 10% PhiX control library. Sequencing of RNA libraries was performed on a NovaSeq 6000 instrument (20,012,850, Illumina, San Diego, CA, USA) utilizing an S4 flowcell and 150-base, paired-end reads (20,028,312, Illumina) with 1% PhiX control library (FC-110–3001, Illumina).

#### Infinium MethylationEPIC array processing

DNA from FFPE tissue and FFZN tissue was extracted using the previously described method. Prior to shipment to the external laboratory, an aliquot of each sample was processed with the enzymatic conversion module (E7125L, New England Biolabs), with up to 200 ng of input. In addition to the enzymatic-converted DNA, unconverted DNA from the same samples was reserved. Both the enzymatic-converted and unconverted DNA samples were then shipped to a contract research organization laboratory for all further processing. All unconverted DNA, derived from both FFPE tissue and FFZN tissue, was bisulfite-treated using the EZ DNA Methylation Kit (D5002, Zymo Research) with 250 ng of input, following the manufacturer’s protocol. The enzymatic- and bisulfite-converted FFPE-derived DNA was then repaired with the FFPE DNA Restore kit (WG-321–1002, Illumina), following the manufacturer’s protocol. Input into the FFPE DNA Restore kit was up to 200 ng for enzymatic-converted samples and up to 250 ng for bisulfite-converted samples. All samples were then processed in one batch for the Infinium MethylationEPIC v1.0 array (WG-317–1003, Illumina), following the manufacturer’s protocol. Samples in Arm 1 were prepared with 3 replicates, Arm 2 were prepared with 6 replicates, and Arm 3 were prepared with 3 replicates.

## Bioinformatics methods

### Methylation sequencing

#### Alignment and CpG extraction

Paired-end sequence data were trimmed and filtered using Trim-Galore v0.6.6 (Babraham Bioinformatics, Cambridge, UK). We optimized hard clip parameters for 5’-Read1, 3’-Read1, 5’-Read2, and 3’-Read2 based on M-bias plots from the Bismark [53] pipeline as follows: bisulfite: 10, 10, 19, 5 and EM-seq: 5, 5, 11, 5, respectively. Alignment to GRCh38.86 and lambda phage J02359.1 was performed using Bismark v0.23.0 with Bowtie2 v2.4.2 [49 = 54] end-to-end mapping implemented in bcbio methylation analysis pipeline (https://bcbio-nextgen.readthedocs.io/en/latest/contents/methylation.html). Read deduplication and CpG extraction were performed with Bismark, and CpGs that were overlapping from paired-end reads were only extracted from Read 1. Usable reads were determined by the number of uniquely mapped, deduplicated reads that were used for CpG methylation extraction.

#### Alignment optimization

Read alignment optimization was performed on 1 million subsampled paired-end reads. Bismark v0.23.0 end-to-end mapping parameters included –score_min values of L,0,-0.2, L,0,-0.4, and L,0,-0.6 as well as –local. For comparison, reads were aligned after trimming using bwa-meth v0.2.2 [55] and CpGs were extracted using MethylDackel v0.5.0 [56] before aggregation. If not specified, maximum insert size thresholds were set to 500.

#### CpG aggregation

Count data from methylated and unmethylated cytosines in CpG context were aggregated from either Bismark CpG_report.txt.gz or MethylDackel bedgraph.gz outputs. Promoter region counts were identified using Ensembl annotations from protein coding transcripts with transcript support level 1. Promoter regions were defined as ± 2 kb around the TSS. CpG islands and RepeatMasker v4.0.9 [57] regions were identified using University of California, Santa Cruz (UCSC) annotations for hg38. Panel results were aggregated according to the capture panel regions. All regions had a minimum 10 coverage filter. Bigwig files were generated using pyBigWig v0.318 [58] after collapsing counts to the fraction of methylated reads per CpG and filtering CpGs with less than 10 combined reads. Pairwise Pearson correlations were used to compare average methylation values across regions between different methylation assays.

### Coverage normalization

Fraction of normalized coverage was determined using CollectGcBiasMetrics from Picard v2.23.3. CpG Island normalized coverage represents the fraction of unique cytosine counts in a particular CpG Island to the total cytosine counts in all CpG Islands.

#### Targeted methylation panel design

The custom-designed Twist Bioscience DNA methylation panel (chosen as it has been optimized to work with EM-Seq) was designed to capture CpG islands within promoters of cancer-driver genes using OncoKB (https://www.oncokb.org/cancerGenes) [59]. Cancer genes were prioritized by frequency of occurrence in MSK-IMPACT [60], MSK-HEME [61], Foundation One [62], Foundation One Heme [63], and Vogelstein cancer genes [64] and Sanger cancer gene census [65], with most selected genes occurring in 3/6 lists (yielding 467 cancer genes). Next, hg38 gene promoters (Gencode v32) ± 10 kb from TSS and the hg38 annotated coordinates of CpG islands were exported using the UCSC table browser. Promoter regions were filtered to the cancer-driver gene list and intersected with UCSC hg38 CpG island annotations using Bioconductor IRanges subsetByOverlaps function in R [66] (at least 1-bp overlap in a non-stranded intersection). Additional capture regions were added for several genes including *BRCA1* with sequence added near *BRCA1* TSS. During the design optimization process, repetitive elements were identified and excluded from the final design. These removed low-complexity regions represented a small fraction (< 1%) of the originally designed area. The final baited region covered 564 regions, totaling 593 kb of captured content.

#### Differential methylation and pathway analysis

Differentially methylated TSS regions (MANE select v0.95) on clinical samples were identified using edgeR v.3.32 [67] glmLRT with Benjamini–Hochberg-corrected *P*-values from methylated and unmethylated count data. Methylated and unmethylated read counts were used to generate a methyl model matrix with matched library sizes and sample as covariate term. Supplementary differential methylation of CpG sites was performed using methylKit v1.16 [68] using logistic regression with F Test, McCullagh/Nelder (MN) overdispersion correction, and the SLIM method for pvalue correction. For progressed versus non-progressed comparisons, age and sex were added as covariates. For pre- versus post-treatment, the patient was used as a covariate. Corrected *P*-values ≤ 0.05 were considered statistically significant. R(v4.0.3). The Biocarta knowledgebase was used to determine what pathways *IL15* expression could be impacting, using a Pearson correlation of expression of *IL15* and all Biocarta pathways. Three differential methylation methods were attempted for Arm3 samples: 1) EdgeR as described above, 2) methylKit as described above, and 3) a MannWhitney U (scipy v1.10) test with Benjamini/Hochberg FDR correction (statsmodels v1.12).

#### EPIC array methods

EPIC array IDAT files were processed using the minfi bioconductor package v1.36 [69]. Raw beta values were normalized according to the ssNOOB method [70]. For comparisons to next-generation sequencing experiments, ssNOOB-adjusted beta values for probes spanning genomic regions of interest were averaged. The GCT (Green CpC to TpC) score is the ration of the mean green signal in CpC probes over the mean green signal in TpC probes and is used to assess conversion efficiency where deviation from 1 is considered incomplete conversion [71]. Failed (p > 0.01) and cross-reactive probes [72] were filtered prior to analysis.

#### ENCODE data processing

ENCODE samples were reprocessed from raw fastq files according to the bioinformatic methods described above (Bismark and bcbio). Fastq files were obtained from ENCODE experiments ENCSR765JPC (GSE86747) and ENCSR890UQO (GSE86765).

#### Significance testing and heatmap generation

All significance testing was performed using scipy v1.10. Group testing was performed using two-sided independent t-test unless otherwise stated. WGMS correlation heatmaps were generated by calculating pairwise Pearson correlations among all common CpG sites with at least 10X coverage (using scipy v1.10, stats.pearsonr, two-sided). The correlation matrices were visualized as heatmaps with seaborn (clustermap), applying hierarchical clustering with average linkage and Euclidean distance. Correlations between Panel and Panel/WGMS was generated using aggregated methylation across panel capture regions with regions with greater than 10X coverage using average linkage and Euclidean distance. Benjamini/Hochberg FDR-corrected pvalues (q values) were generated using statsmodels v0.13.5. 95% Confidence Intervals, referred to as 95% CI, are nonparametric and are generated using seaborn v0.12.2 by taking a 95 percentile interval of the bootstrap distribution.

#### Principal component analysis (PCA)

Prior to PCA, a common set of CpG Island regions that were covered by WGMS, Panel and EPIC were selected for analysis. Regions with coverage less than 10 in WGMS or Panel were filtered. Average methylation across these regions was aggregated by read for WGMS and Panel, and by normalized beta value for EPIC. PCA was performed on the combined dataset using scikit learn (v1.1.3).

### RNA sequencing

#### Expression quantification

Paired-end fastq files were processed using the bcbio-nextgen pipeline (https://bcbio-nextgen.readthedocs.io/en/latest/contents/bulk_rnaseq.html) for RNA-seq. Transcripts Per Kilobase Million (TPM) values were generated using salmon v1.4.0 using Ensembl gene build 94 annotations and GRCh38_full_analysis_set_plus_decoy_hla genome fasta from The International Genome Sample Resource (IGSR).

## Supplementary Information

Additional file 1.

## Data Availability

The datasets generated and/or analyzed during the current study are available in the Sequence Read Archive (SRA) under SRA submission ID SUB15578058. The CLL methylation datasets described in this manuscript may be obtained in accordance with AstraZeneca’s data sharing policy described at https://astrazenecagrouptrials.pharmacm.com/ST/Submission/Disclosure.—Data for studies directly listed on Vivli can be requested through Vivli at www.vivli.org. Data for studies not listed on Vivli could be requested through Vivli at https://vivli.org/members/enquiries-about-studies-not-listed-on-the-vivli-platform/. AstraZeneca Vivli member page is also available outlining further details: https://vivli.org/ourmember/astrazeneca/.—Documentation and source code files for the analyses methods used are freely available from the authors on request.

## Abbreviations

5hmc: 5-Hydroxymethylcytosine
5mC: 5-Methylcytosine
BS-seq: Bisulfite cytosine conversion and next-generation sequencing
BTK: Bruton tyrosine kinase
cfDNA: Circulating cell-free plasma DNA (also called circulating tumor DNA [ctDNA])
CLL: Chronic lymphocytic leukemia
CRC: Colorectal cancer
EM-seq: Enzymatic cytosine conversion and next-generation sequencing
FFPE: Formalin-fixed paraffin-embedded
FFZN: Fresh frozen
NSCLC: Non-small cell lung cancer
PBAT: Post-bisulfite adapter tagging
PBMC: Peripheral blood mononuclear cells
PCR: Polymerase chain reaction
TSS: Transcriptional start site
WGMS: Whole genome methylation sequencing
