# Supplementary material for: Comprehensive comparison of enzymatic and bisulfite DNA methylation analysis in clinically relevant samples
Source: Clin Epigenetics. 2025 Oct 3;17:156. doi: 10.1186/s13148-025-01959-0 (PMC12495756; doi:10.1186/s13148-025-01959-0)

# SUPPLEMENTAL MATERIAL

**Supplemental Table 1.** Sequencing summary statistics.

| Arm | Samples | Conversion Methods | Unique Number of Samples | Replicates per Sample | Total Number of Libraries | Average Paired End Reads (M) | Average WGMS Coverage |
| --- | --- | --- | --- | --- | --- | --- | --- |
| #1 | HCT116 Titration | Enzymatic | 5 | 3 | 15 | 359 | 26.8 |
|  |  | Bisulfite | 5 | 3 | 15 | 365 | 27.2 |
| #2 | NA12878 & K562 | Enzymatic | 2 | 6 | 12 | 373 | 27.3 |
|  |  | Bisulfite | 2 | 6 | 12 | 427 | 34.4 |
| #3 | Colon & NSCLC FFPE | Enzymatic | 3 | 3 | 9 | 289 | 19.8 |
|  |  | Bisulfite | 3 | 3 | 9 | 417 | 34.4 |
| #3 | Colon & NSCLC FF | Enzymatic | 6 | 3 | 18 | 287 | 20.6 |
|  |  | Bisulfite | 6 | 3 | 18 | 382 | 31.5 |

**Supplemental Table 2.** Arm 2 and Arm 3 BS-Seq libraries were 38% and 51% smaller than expected based on the Covaris fragmentation size specified and the final measured average library fragment size respectively, whereas EM-Seq libraries were only 16% and 8% smaller. Arm 3 cfDNA libraries were prepared without additional fragmentation from the same sample and were consistently larger for EM-Seq​ then BS-Seq.

| **​** | **Arm 2​ tissue** | | **Arm 3​ tissue** | | **Arm 3 cfDNA​** | |
| --- | --- | --- | --- | --- | --- | --- |
| ​ | BS-Seq​ | EM-Seq​ | BS-Seq​ | EM-Seq​ | BS-Seq​ | EM-Seq​ |
| Fragmentation size (bp)​ | 350 | 250 | 350 | 250 | 166 | 166 |
| Final library fragment size (bp)​ | 217 | 209 | 173 | 230 | 166 | 187 |
| Difference (bp)​ | -133 | -41 | -177 | -20 | 0 | 21 |
| Percentage difference​ (Obs/Exp) | 38% | 16% | 51% | 8% | 0% | -12% |

**Supplemental Table 3.** Patient-specific data on the timepoints of CLL sample collection.

**Supplemental Figure 1.** WGMS workflows. The BS-Seq method reported is a PBAT protocol, which has been shown to increase the quality of methylation sequencing by reducing the impact of DNA damage caused by bisulfite conversion. EM-Seq (and the bisulfite method reported in Vaisalva 2019) is a post-bisulfite adapter–tagging protocol. BS-Seq, bisulfite cytosine conversion and next-generation sequencing; EM-Seq, enzymatic cytosine conversion and next-generation sequencing; PBAT, post-bisulfite adapter tagging.


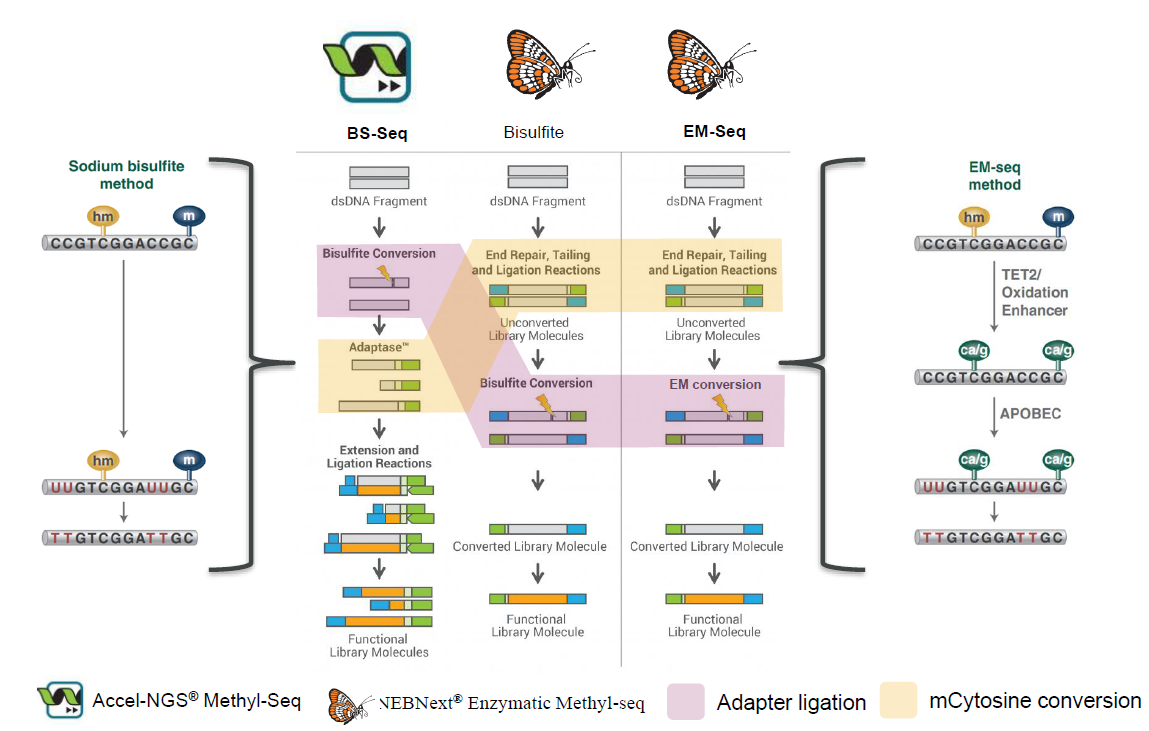


**Supplemental Figure 2.** A) Lambda conversion rate of HCT116 DNMT1 and DNMT3b knockout cell lines diluted with in vivo methylated DNA. B) Lambda conversion rate of NA12878 and K562 sequencing libraries. C) Mapping efficiency of arm 2 cell line libraries. D) Arm 2 average methylation aggregated by repeat class and cell line (*P* = 7.0^e-3^). E) Median fragment size and (F) median read length for arm 2 cell line libraries after M-bias–directed read trimming. G) Library yield and H) duplication rate of arm 2 cell lines by conversion methods (*P* = 2.2^e-5^). BS-Seq, bisulfite cytosine conversion and next-generation sequencing; EM-Seq, enzymatic cytosine conversion and next-generation sequencing.


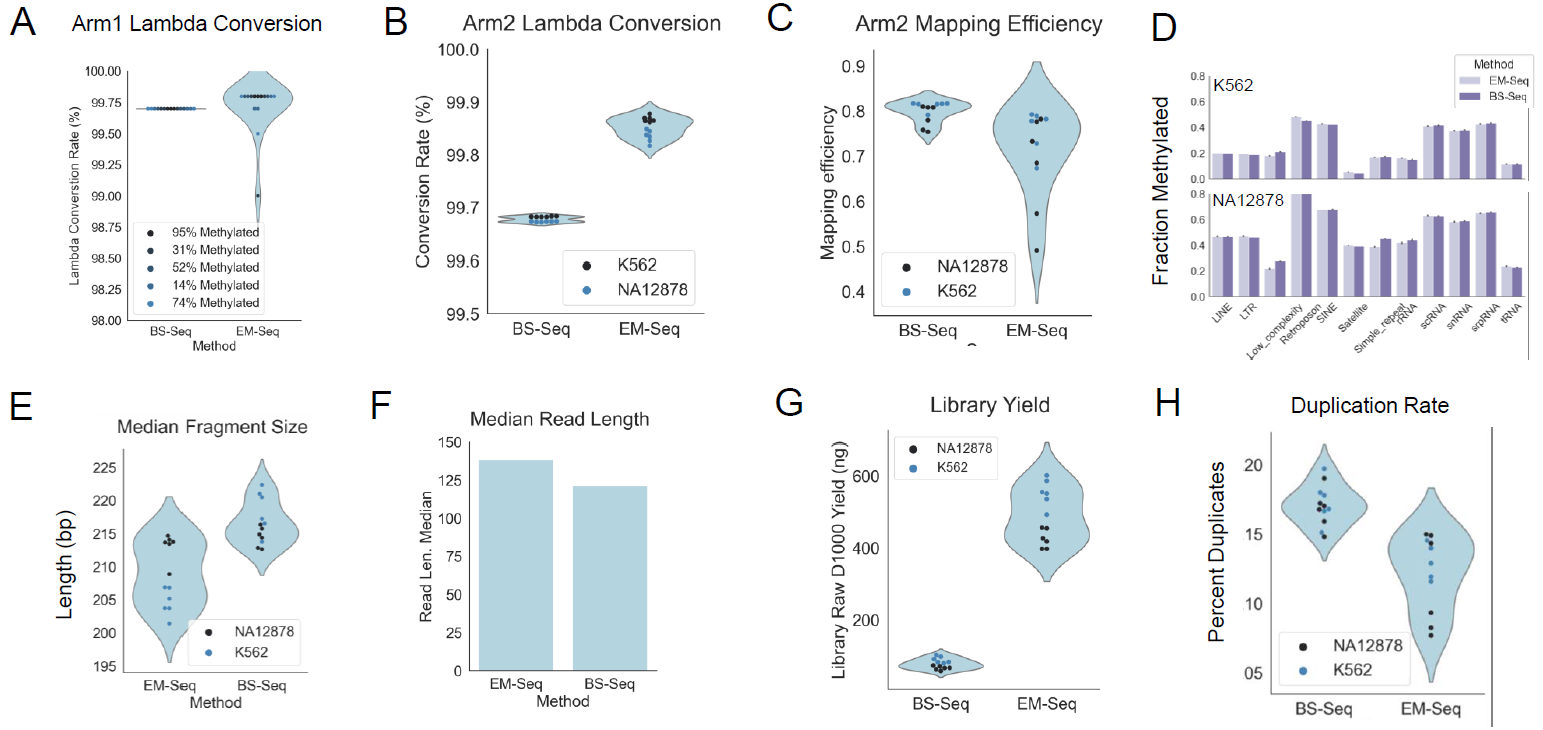


**Supplemental Figure 3.** A) Picard estimated library size for arm 3. B) Average percent mapping of EM-Seq and BS-Seq libraries across select Bismark parameters. C) General error rate from aligned reads in arm 3. D) Results of differential methylation tests for CpG island methylation in arm 3 comparing FFPE vs FFZN. E) Normalized coverage over CpG islands and CpG island density by GC% (95% CI). BS-Seq, bisulfite cytosine conversion and next-generation sequencing; EM-Seq, enzymatic cytosine conversion and next-generation sequencing; FFPE, formalin-fixed paraffin-embedded; FFZN, fresh frozen; GC, guanine/cytosine.


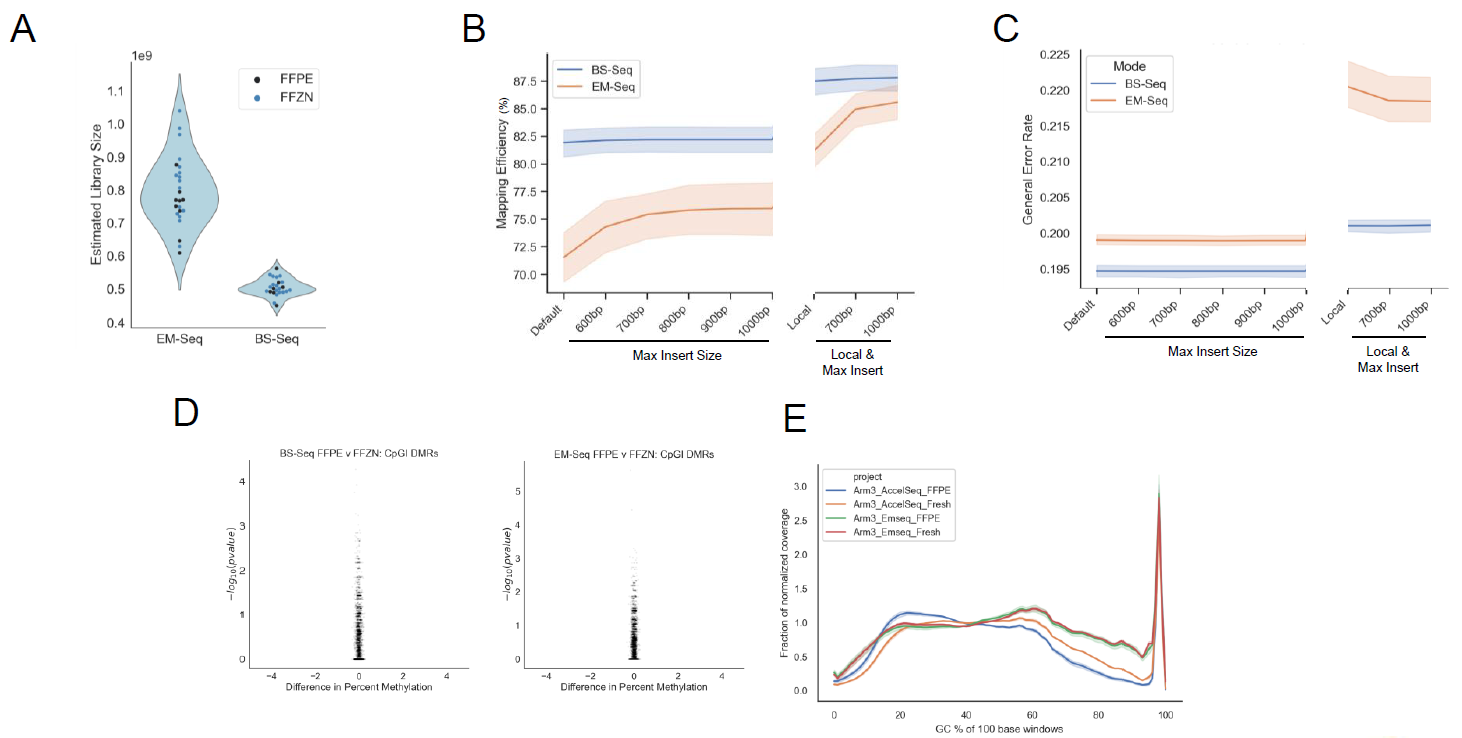


**Supplemental Figure 4.** A) Summary alignment and library statistics for panel capture libraries. B–E) Correlation of methylation across panel capture regions for (B) arm 3 FFPE BS-Seq; (C) arm 3 FFZN BS-Seq; (D) arm 3 FFPE EM-Seq; and (E) arm 3 FFZN EM-Seq. Boxed areas represent grouped panel samples. BS-Seq, bisulfite cytosine conversion and next-generation sequencing; cfDNA, circulating cell-free plasma DNA; EM-Seq, enzymatic cytosine conversion and next-generation sequencing; FFPE, formalin-fixed paraffin-embedded; FFZN, fresh frozen; WGMS, whole genome methylation sequencing.


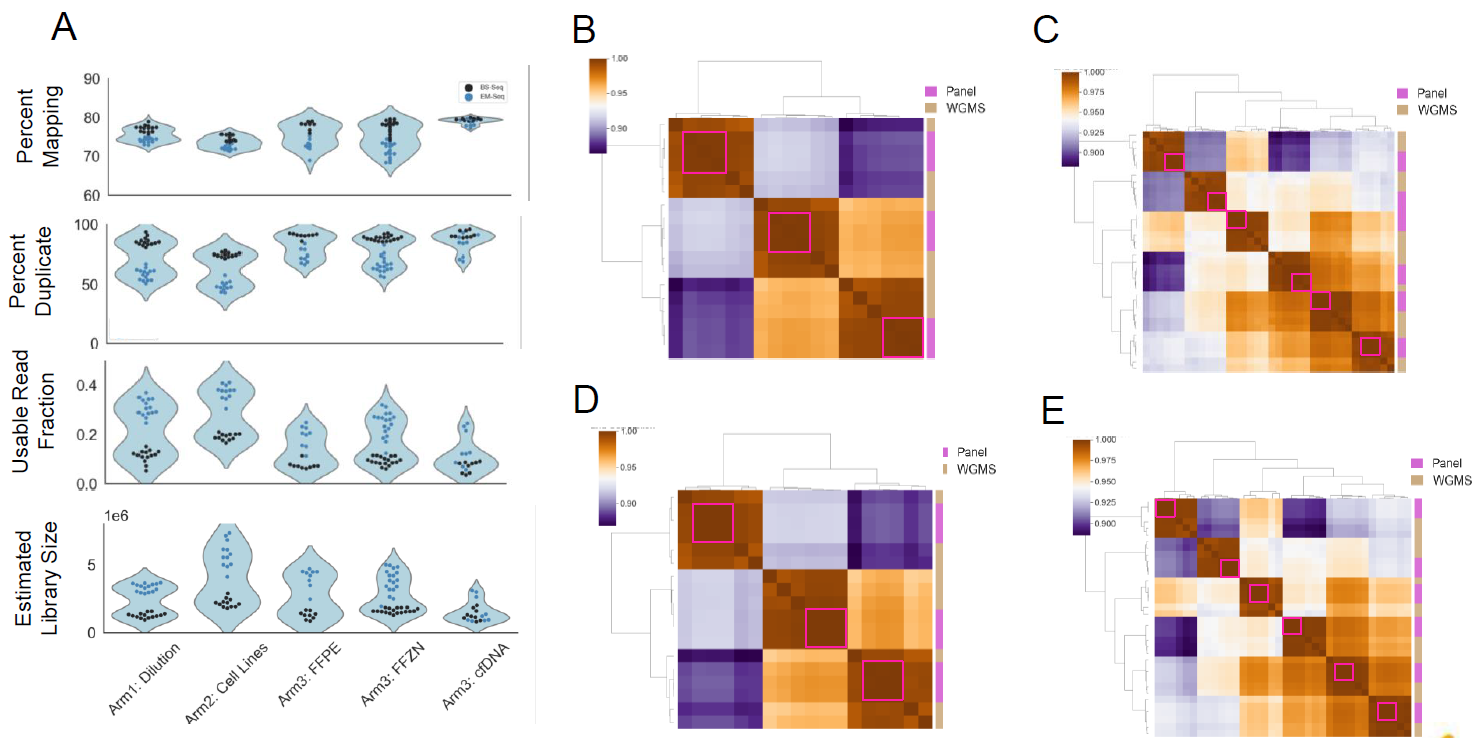


**Supplemental Figure 5.** A) Heatmap of differentially methylated regions between tumor and normal cfDNA in CRC1 and B) CRC2. C) Sample signal from panel capture sequenced arm 3 CRC replicates at the NKX2-1–associated CpG island and D) FOXA1–associated CpG island. E) GCT_1 control conversion values for EPIC arrays grouped by conversion method. F) Correlation of WGMS average methylation and EPIC normalized beta values across aggregated CpG islands for all arm 3 samples. cfDNA, circulating cell-free plasma DNA; CRC, colorectal cancer; EPIC, MethylationEPIC array; PE, formalin-fixed paraffin-embedded; FF, fresh frozen; WGMS, whole genome methylation sequencing.


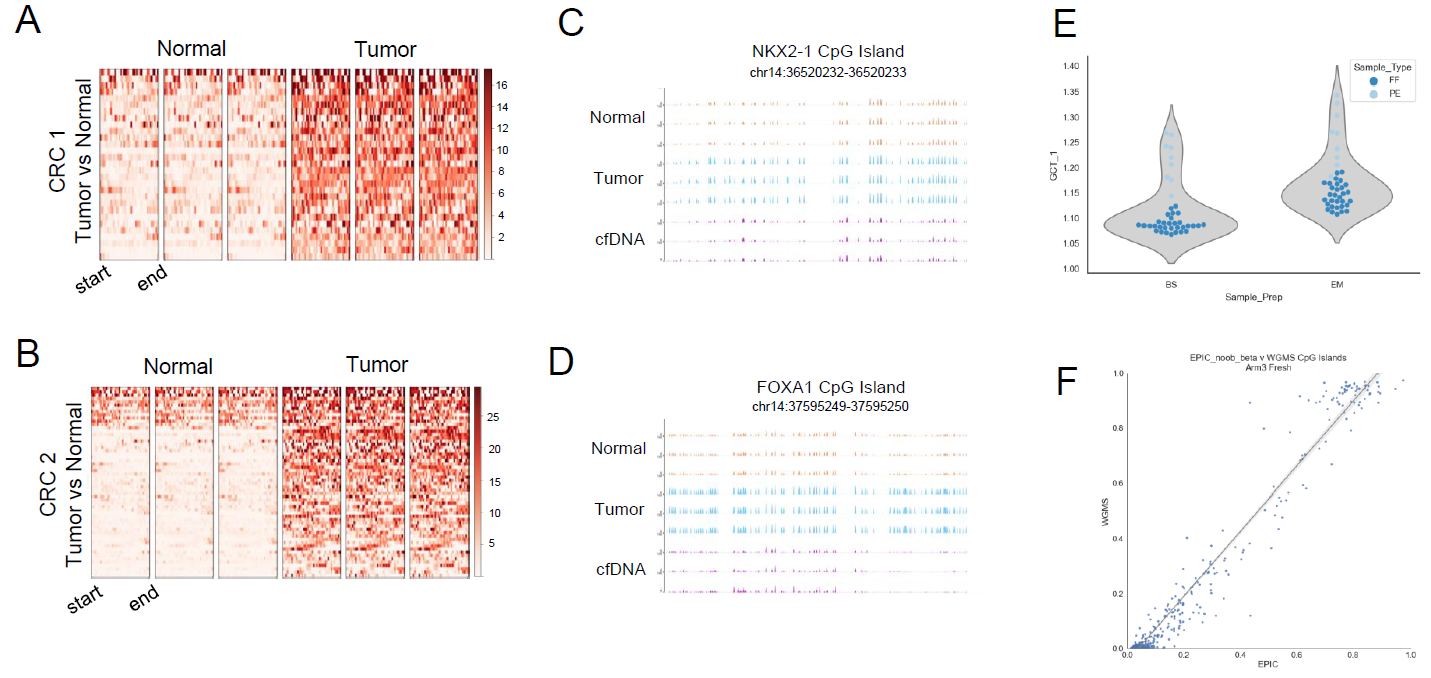


**Supplemental Figure 6.** A) Lambda conversion efficiency. B) Venn Diagram overlapping differentially methylated regions from edgeR and methylKit comparing progressed vs non progressed after treatment. (p<1e-5, Hypergeometic), including IL15. C) Correlation of CDKN1C (*R=*0.07, n.s.) and D) AXL (*R=*-0.32, p=0.01) methylation with expression from bulk RNA-seq data from corresponding samples. E) Distribution of Transcripts Per Million (TPM) counts for CDKN1C in the Progressed and Non-Progressed patient populations (ns, p>0.01). E) Distribution of Transcripts Per Million (TPM) counts for AXL in the Progressed and Non-Progressed patient populations (ns, p>0.01)


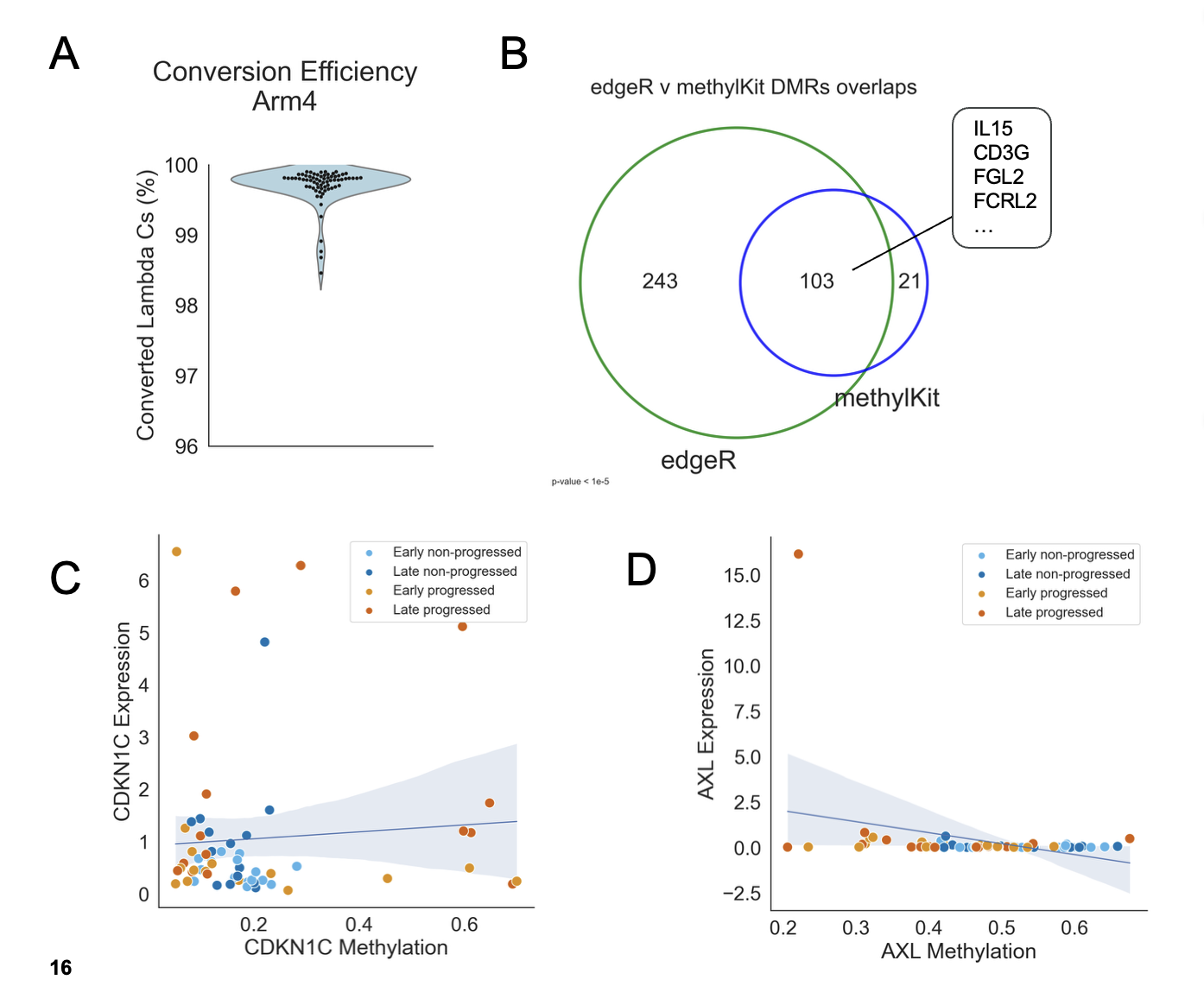

Supplement: Supplementary file 1 — Additional file 1. [file 13148_2025_1959_MOESM1_ESM.docx]
